# Impacts of manure application on soil environment, rainfall use efficiency and crop biomass under dryland farming

**DOI:** 10.1038/srep20994

**Published:** 2016-02-12

**Authors:** Xiaojuan Wang, Zhikuan Jia, Lianyou Liang, Baoping Yang, Ruixia Ding, Junfeng Nie, Junpeng Wang

**Affiliations:** 1 China Water-saving Agricultural Academy in Arid Areas, Northwest A&F University, 712100, Yangling, Shaanxi, PR China; 2 Institute of Dryland Farming, Shanxi Academy of Agricultural Sciences, 030038, Taiyuan, Shanxi, PR China; 3 College of Resources and Environment, Northwest A&F University, 712100, Yangling, Shaanxi, PR China

## Abstract

Because of inadequate nutrient and water supply, soils are often unproductive in Northwest China. We studied the effects of manure application at low (LM 7.5  t ha^–1^), medium (MM 15 t ha^–1^), and high (HM 22.5 t ha^–1^) rates combined with fixed levels of chemical fertilizers on maize growth and rainfall use efficiency compared with chemical fertilizers (CK) under semi-arid conditions over a three-year period. HM and MM treatments could significantly increase soil water storage (0–120 cm) at tasseling stage of maize compared with LM treatment and CK (*P *< 0.05). Dry matter accumulation and rainfall use efficiency increased as manure application rate increasing (*P *< 0.05). HM treatment significantly increased rainfall use efficiency by 6.5–12.7% at big trumpeting – tasseling stage compared with LM and MM treatments. HM and MM treatments increased rainfall use efficiency by 8.6–18.1% at tasseling – grain filling stage compared with CK. There was no significant difference on biomass between HM and MM treatments at grain filling and maturity stages of maize in 2009 and 2010.

The most limiting factors for crop production are water and soil nutrient in semiarid areas of China. Dryland farming is practiced on about one third of the arable land of China, of which about 40% is situated on the Loess Plateau of China^1^. The climate of the Loess Plateau is mostly semiarid, with annual precipitation ranging from 150–300 mm in the north to 500–700 mm in the south^2^. Rainfall distribution is uneven, with 60–70% of the rainfall during July-September, meanwhile, soil water evaporation is high in the Loess Plateau region of China^3^. More than 90% of the cropland in this area receives no irrigation. As the second main crop in the Loess Plateau region, maize was conventionally cultivated with a single crop being produced per year, followed by about seven months fallow. In the fallow period, water can be stored in the soil and used by the subsequent maize crop. Both fertilizer applications and crop yields have increased in the past 20 years, further depleting soil water^4^. Therefore, a dry subsoil layer has formed and the crop yield varies strongly with rainfall in the growing season^5^. Available water for plant in the fallow period may replenish the dry soil layer if sufficient rain falls to penetrate the soil profile^6^. However, the conventional practice of keeping the soil water only during the fallow period resulted in very low fallow efficiency (ratio of stored water to rainfall during fallow)^7^,^8^. In addition, the infiltration depth is shallower in high fertilization than low fertilization conditions^9^. Therefore, the conventional practice with high fertilization does not appear to be a sustainable management option in the long-term. There is a need to develop technologies that optimize the use of the limited water and soil resources to achieve sustainable crop production. Rational application of organic manure has been shown to improve the water infiltration, water retention^10^, soil water contents^11^,^12^,^13^, grain yield^14^,^15^,^16^,^17^, and rainfall use efficiency (dry matter accumulation/precipitation, in kg ha^−1^mm^−1^)^18^. The importance of fertilization to optimize the use of stored water in the root zone has been recognized^19^,^20^. Farmyard manure combined with inorganic fertilizers plays an important role in better penetrations^21^ and establishment of crop roots^22^. The better roots help the plant to utilize water from deeper layers and to maintain higher relative plant water content under a soil moisture stress condition, which is quite common in rain-fed farming^21^. Additionally, such integration is recognized as a fundamental approach to improve efficient rainfall use for crop production^18^. Meanwhile, the challenge for Northwest China is low rainfall use efficiency, resulting from lower and erratic precipitation, and unbalanced fertilization^23^. However, unreasonable use of manure may have adverse influences on water and soil contamination. Edmeades^24^ showed that inappropriate use of manure induced soil nitrogen (N) and phosphorus (P) leaching. Therefore, it is need to identity the rational manure application rate for promoting the stable and sustainable development of crop production.

The present research is undertaken to study the effects of three rates of manure combined with the same inorganic fertilizer on dry matter accumulation and rainfall use efficiency of maize at different growth stage of maize in a semi-arid agro-ecosystem. The aim of the research is to produce scientific support for giving useful guidelines for farmers of semi-arid area on how to optimize agro-management practices for saving water, rainfall use efficiency, and high-yield maize cultivation.

## Results

### Soil water storage and soil nutrient

The change of soil water storage at different growth period of maize varied in different treatments during three experimental years (Table 1). Manure treatments could restrain soil water evaporation in sowing – jointing stage, but not significant. At jointing – big trumpet stage of maize, temperature rose gradually and water evaporation from soil surface increased daily which resulted in the increasing of water consumption of maize. As fertilization year increased, HM and MM treatments significantly increased soil water storage in big trumpet stage of maize compared with LM treatment and CK and the soil water storage significantly increased as manure application rate increased (*P *< 0.05). In 2009 and 2010 respectively, HM treatment resulted in 5.6% and 14.2% higher soil water storage compared with LM treatment, and 7.5% and 14.9% higher soil water storage compared with CK in big trumpet stage of maize. HM treatment increased soil water storage by 8.9% compared with CK, and MM treatment increased it by 4.8% and 5.5% compared with LM treatment and CK respectively in 2010. The results indicated that increasing the manure application rate could significantly improve soil water condition at big trumpet stage of maize as fertilization year increased (*P *< 0.05).

HM treatment increased soil water storage by 5.9–13.6% compared with LM treatment, and MM treatment increased it by 4.8–12.8% and by 5.0–16.0 compared with LM treatment and CK respectively in the years of 2008–2010 at tasseling stage of maize. The results indicated that HM and MM treatments could significantly improve soil water condition at tasseling stage of maize (*P *< 0.05).

Compared with CK, HM treatment increased soil water storage by 22.6% and 12.5%, while MM treatment increased it by 13.9% and 7.4% at grain filling stage of maize in the years of 2008 and 2009 respectively. In the years of 2008 and 2009 respectively, HM treatment increased soil water storage by 7.6% and 4.8% compare with MM treatment, and by 19.4% and 10.7% compared with LM treatment, while MM treatment increased it by 10.8% and 5.6% compare with LM treatment at grain filling stage of maize. Soil water storage with manure treatments were not significantly higher than that with CK and the soil water storage increased as manure application rate increased at grain filling stage of maize in 2009. The results indicated that increasing the manure application rate could improve soil water condition at grain filling stage of maize. There was no significant difference in soil water storage among different treatment in maturity stage of maize.

**Figure 1:**
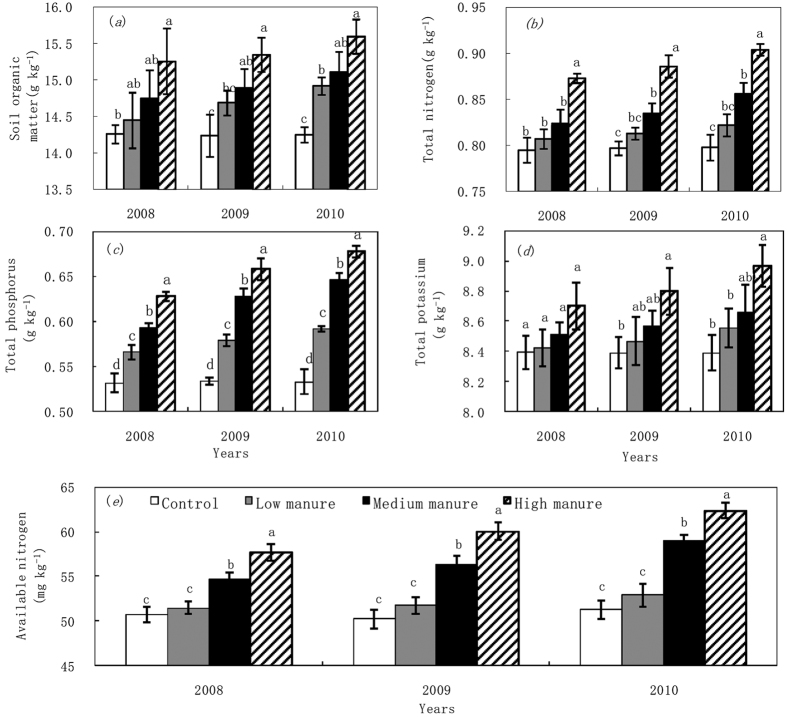
Soil nutrients of (**a**) soil organic matter, (**b**) total N, (**c**) total P, (**d**) total K and (**e**) available N as a function of the different manure treatments during 2008–2010. Bars with the same letter for the same year are not significantly different at *P *= 0.05.

The effect of manure application on soil nutrients is shown in Fig. 1. Compared to CK, all manure treatments significantly increased SOM and total N as fertilization years increased (*P* < 0.05). Manure treatments resulted in higher total P with 6.4–27.2% compared with CK. Total P increased significantly as manure application rate increased. There had slightly increase of total K in LM and MM treatments compared with CK, while HM treatment increased soil total K by 4.9% in 2009 and by 6.9% in 2010 compared with CK. Total K and SOM slightly increased as manure application rate increased. Over the course of the experiment, the amount of SOM, total N, total P and total K was almost stable with CK, whereas these tended to increase with all the manure treatments. MM and HM manure treatments significantly increased the available N content compared with CK (*P *< 0.05). The HM treatment significantly increased the available N compared with LM and MM treatments (*P *< 0.05). The MM treatment significantly increased the available N compared with LM treatment (*P *< 0.05).

### Dry matter accumulation

Manure application significantly increased dry matter accumulation at big trumpet - maturity stages of maize in varying degrees compared with CK (*P *< 0.05) (Table 2). Compared with CK, LM treatment increased dry matter accumulation by 12.6–45.8% at big trumpet stage of maize, and by 11.6–21.4% at tasseling stage of maize, and by 4.6–8.3% at grain filling stage of maize, and by 9.0–14.6% at maturity stage of maize. While MM treatment increased it by 23.1–69.9% at big trumpet stage of maize, and by 20.6–27.1% at tasseling stage of maize, and by 17.1–20.4% at grain filling stage of maize, and by 20.0–29.5% at maturity stage of maize in 2008–2010. In addition, HM treatment increased dry matter accumulation by 38.7–91.5% at big trumpet stage of maize, and by 30.3–38.6% at tasseling stage of maize, and by 22.6–26.8% at grain filling of maize, and by 26.5–37.4% at maturity stage of maize in 2008–2010. The results indicated that manure application could significantly increase dry matter accumulation at big trumpet - maturity stages of maize (*P* < 0.05).

Compared with LM treatment, MM treatment increased dry matter accumulation by 6.9–16.5%, 4.7–8.1%, 10.7–14.2%, 9.0–16.0% in 2008–2010 at big trumpet, tasseling, grain filling and maturity stages of maize respectively. While HM treatment increased dry matter accumulation by 23.5–31.4%, 14.0–16.7%, 17.0–17.6%, 16.1–21.2% in 2008–2010 at big trumpet, tasseling, grain filling and maturity stages of maize respectively. In big trumpet and tasseling stages of maize respectively, HM treatment increased dry matter accumulation by 8.1–10.4%, 11.3–12.7%, and 8.0–9.1% in 2008–2010 compared with MM treatment. The results indicated that increasing manure application rate could significantly increase dry matter accumulation at big trumpet and tasseling stages of maize (*P *< 0.05).

At grain filling and maturity stages of maize respectively, HM treatment increased dry matter accumulation by 6.2% and 10.0% compared with LM treatment, while there was an insignificant increase in 2009 and 2010.

### Rainfall use efficiency

Manure application improved rainfall use efficiency at big trumpet - maturity stages of maize in varying degrees (Table 3). Rainfall use efficiency with manure treatments was significantly higher than that with CK at jointing – tasseling stage and grain filling – maturity stage of maize, and rainfall use efficiency significantly increased as manure application rate increased at grain filling – maturity stage of maize (*P *< 0.05). Compared with CK, LM treatment increased rainfall efficiency by 13.5–50.6% at jointing – big trumpet stage of maize, and by 4.8–23.0% at big trumpet – tasseling stage of maize, and by 11.0–45.4% at grain filling – maturity stage of maize. While MM treatment increased it by 24.6–77.2% at jointing – big trumpet stage of maize, and by 10.91–28.1% at big trumpet – tasseling stage of maize, and by 18.65–77.67% at grain filling – maturity stage of maize in 2008–2010. In addition, HM treatment increased it by 41.20–100.95% at jointing – big trumpet stage of maize, and by 18.14–38.54% at big trumpet – tasseling stage of maize, and by 25.78–96.68% at grain filling – maturity stage of maize in 2008–2010. The above indicated that manure application could significantly increase rainfall use efficiency in jointing –tasseling and grain filling – maturity stages of maize (*P *< 0.05).

Compared with LM treatment, MM treatment increased rainfall use efficiency by 7.2–17.7% at jointing – big trumpet stage of maize, and by 4.6–22.2% at grain filling – maturity stage of maize in 2008–2010. While HM treatment increased it by 21.5%- 33.5% at jointing – big trumpet stage of maize, and by 13.3–35.3% at grain filling – maturity stage of maize in 2008–2010. HM treatment increased rainfall use efficiency by 11.9–33.5% at jointing – big trumpet stage of maize, and by 6.0–20.2% at grain filling – maturity stage of maize compared with MM treatment. The results indicated that increasing manure application rate could significantly increase rainfall use efficiency at jointing – big trumpet and grain filling – maturity stages of maize (*P *< 0.05).

MM treatment resulted in 5.81% higher rainfall use efficiency at big trumpet – tasseling stage of maize in 2010. Compared with CK, MM treatment increased rainfall use efficiency by 8.6–18.1%, and HM treatment increased it by 12.7–13.6% at tasseling –grain filling stage of maize.

It could be seen from above that manure application could increase rainfall use efficiency and achieve the aim of more efficient use of rainfall at different growth period of maize.

**Figure 2:**
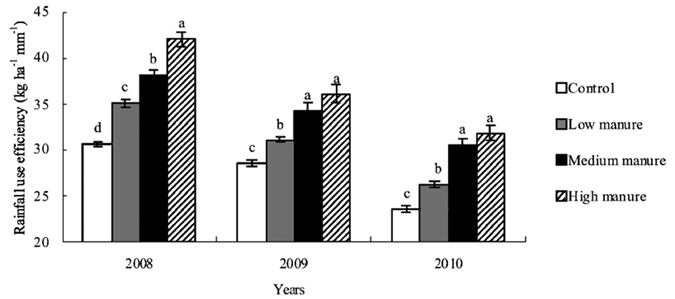
Effect of manure management on rainfall use efficiency in whole growth period of maize. Bars with the same letter for the same year are not significantly different at *P *= 0.05.

Manure application could significantly increase rainfall use efficiency in the whole growth period of maize (*P* < 0.05) (Fig. 2). Compared with CK, LM treatment increased rainfall use efficiency by 9.0–14.6%, and MM treatment increased it by 20.0–29.5%, and HM treatment increased it by 26.5–37.4% in the whole growth period of maize in 2008–2010. Compared with LM treatment, MM treatment increased rainfall use efficiency by 9.0–16.0%, and HM treatment increased it by 16.1–21.2% in 2008–2010. Rainfall use efficiency with HM treatment was 10.0% higher than that with MM treatment in 2008, while in 2009 and 2010 it was not significantly higher than that with MM treatment. The results indicated that as fertilization year increased there was no significant difference in rainfall use efficiency between MM treatment and HM treatment.

## Discussion

Wang^25^ *et al.* found an increase of soil water in fertilizer to no-fertilizer management. In this research, HM and MM treatments significantly increased soil water storage at big trumpet, tasseling and grain filling stages of maize (*P *< 0.05). This increase was probably because the application of high or medium rate of manure increased soil total porosity and aggregate content^26^,^27^,^28^, reduced invalid water consumption of crop^25^ and increased rainfall use efficiency (Table 4). HM and MM treatments significantly increased soil water storage compared with LM treatment and CK, and HM treatment significantly increased soil water storage compared with MM treatment in grain filling stage (*P *< 0.05) in the years of 2008 and 2010, while in 2009 there was no significant difference among each treatment. This may be due to the frequently small-scale rainfall between tasseling stage and grain filling stage of maize (July 24 to August 22) in 2009 (Table 1). In 2009, frequently small-scaled rainfall might result in non-significant difference in soil evaporation among manure treatments, while the higher manure application probably consumed more soil water due to the higher dry matter accumulation. Therefore higher manure application resulted in non-significantly higher soil water content compared with lower manure application.

Affholder^29^ indicated that the application of manure had a positive effect on crop dry matter. Li *et al.*^30^ showed that biomass was related to nutrition supply. In this study, the combined use of inorganic fertilizers and organic manures significantly increased dry matter accumulation and dry matter accumulation increased as manure application rate increased at different growth stages of maize. This might because higher manure application rate improved soil physical^27^,^31^,^32^, chemical^15^,^26^,^33^,^34^,^35^ and biological^36^,^37^,^38^ properties and made roots extended deeper in the environment of adequate nutrient supply^39^. Increased root proliferation increased the volume of soil colonized, thereby reduced the probability of plant growth being restricted by intermittent periods of drought^30^. HM treatment significantly increased dry matter accumulation in grain filling and maturity stages of maize compared with LM treatment in 2008 (*P *< 0.05), while in 2009 and 2010 there was a slight increase. This may be because of different intensity and timing of rainfall in different year.

In present study, the combined use of inorganic fertilizers and organic manure enhanced rainfall use efficiency and the rainfall use efficiency increased as manure application rate increased. This might because higher manure application rate increased soil nutrient^26^,^40^,^41^ and then increased crop biomass^39^. MM treatment significantly increased rainfall use efficiency in whole growth period of maize compared with chemical fertilizers treatment in 2008 (*P *< 0.05), while in 2009 and 2010 there was a slight increase. This may be due to the different intensity and timing of rainfall in different year. MM treatment slightly increased rainfall use efficiency in big trumpet- tasseling stage of maize compared with LM treatment in 2008 and 2009, while in 2010 there had a significant increase (*P *< 0.05). This might because of the lower rainfall in 2008 and 2009 (27 mm and 46.6 mm respectively) compared with the rainfall in 2010 in big trumpet- tasseling stage of maize (92.4 mm).

## Conclusions

Higher manure application amount had a positive effect on developing production potential of rainfall resource, and could improve soil water condition and increase rainfall use efficiency at each growth period of crop to obtain the goal of efficient utilization of rainfall resource, which laid foundations for increasing crop biomass yield. The best manure application rate for improving crop dry matter accumulation and rainfall use efficiency was 15t ha^–1^.

## Materials and Methods

### Site description and experimental design

A three-year field experiment with maize was conducted on dark loessial soil (sand 26.83%, silt 41.91%, and clay 21.03%) between 2008 and 2010 at the Ganjing Research Station of the Northwest A&F University, Heyang, Shaanxi China (35°24′N, 110°17′E; 850 m altitude). The mean annual temperature was 9–10 °C. The experimental site is characterized by low and erratic rainfall with droughts occurring at different stages of maize growth. The long-term mean annual rainfall at the site was 571.9 mm and the mean annual evaporation was 1832.8 mm. Most of the rainfall occurred from July to September. In the years from 2008–2010, the rainfall during the maize growth period was 350.8, 379.1, and 422.3 mm, respectively (Table 4). Based on an analysis of soil samples taken from the experimental area in October 2007, the top 20 cm of soil had the following characteristics: pH 8.1, soil organic matter 14.3 g kg^–1^, total N 0.8 g kg^–1^, total P 0.5 g kg^–1^, total potassium (K) 8.4 g kg^–1^.

The field experiment used a completely randomized block design with four treatments, three replicates, and a 4 × 6 m plots. The four treatments were as follows: application of chemical fertilizers only (CK); application of manure at 7.5 (LM), 15 (MM), 22.5 (HM) t ha^–1^ in combination with chemical fertilizer. The N and P content of the chemical fertilizers were 255 kg ha^–1^ and 90 kg ha^–1^, respectively. The N and P fertilizers were applied separately as basal fertilizers before sowing the maize, at rates of 102 kg N ha^–1^ and 90 kg P ha^–1^, respectively. Additional N fertilizer was applied at a rate of 153 kg N ha^–1^ during the stage when maize had a spear-shaped top (late July). Chicken manure was used, which contained 12.6 g kg^–1^ N, 6.4 g kg^–1^ P, and 13.4 g kg^–1^ K. The manure was applied in each experimental year after maize harvesting at the end of September. The maize variety was Shendan 16. In each experimental year, maize was planted at a rate of 49500 plants ha^–1^ in mid-April and was harvested in mid-September. No irrigation was provided in any of the experimental years.

### Sampling and analysis methods

Soil water content was measured gravimetrically (drying method, w/w) to a depth 120 cm at 20 cm increments before sowing and at different growth stages of maize. Three random locations in each plot were taken to measure soil water content. The soil bulk density was determined according to Robertson *et al.*^42^.

Soil water storage (0–120 cm) was calculated using the formula:


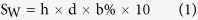


where S_W_ (mm), the sum of soil water storages at different soil layers; h (cm), soil layer depth; d (g cm^−3^), soil bulk density in different soil layer and b%, the percentage of soil moisture in weight.

Dry matter was measured at different growth stages of maize. All samples of maize were dried in an oven at 105 °C for 1 h and then were dried at 75 °C to constant weight. Five corn plants per plot were used (destructively sampled) for each measurement at different growth stages of maize.

Rainfall use efficiency was calculated as the following formula^43^:


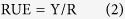


where RUE represents the rainfall use efficiency for the biomass yield (kg ha^−1^mm^−1^); Y is the dry matter accumulation of the maize, and R is the rainfall.

Soil samples were collected from the surface layers (0–20 cm) of all plots immediately after the maize harvest during September each year. Five soil samples were collected for each treatment replicate, and they were combined into a single composite sample. The soil samples were air-dried and were sieved before chemical analysis. All chemical parameters were calculated based on the oven-dry (105 °C) weight of the soil.

Soil organic matter (SOM) was determined using the dichromate oxidation method^44^, total N by micro-Kjeldahl digestion, total P was determined by the wet oxidation procedure described by Rowland and Grimshaw^45^; and total K by extraction with 1N ammonium acetate (NH_4_OAc) solution at pH 7.0^46^.

### Statistical analysis

Repeated-measures analysis of variance (ANOVA) was performed using the program SAS 6.2 for Windows. The significance of treatment effects was determined using the F-test. Multiple comparisons of means was performed using Duncan’s multiple range test^47^ at the *P* ≤ 0.05 level.

## Additional Information

**How to cite this article**: Wang, X. *et al.* Impacts of manure application on soil environment, rainfall use efficiency and crop biomass under dryland farming. *Sci. Rep.* **6**, 20994; doi: 10.1038/srep20994 (2016).

## Figures and Tables

**Table 1:** Soil water storage at 0–120 cm soil profile as influenced by manure management.

Years	Treatments	Soil water storage (mm)					
		Sowing	Jointing stage	Big trumpetstage	Tasseling stage	Grain fillingstage	Maturity stage
2008	Control	271.4 ± 6.0a	265.7 ± 6.2a	274.2 ± 4.6a	248.5 ± 2.1b	231.8 ± 5.1c	226.3 ± 2.3a
	Low manure	273.2 ± 5.3a	267.2 ± 4.8a	276.9 ± 3.8a	249.8 ± 2.8b	238.2 ± 4.2c	227.4 ± 3.1a
	Medium manure	275.3 ± 5.6a	269.9 ± 5.1a	279.6 ± 4.1a	261.8 ± 2.6a	264.0 ± 3.7b	228.3 ± 2.6a
	High manure	283.0 ± 7.1a	275.8 ± 5.8a	279.8 ± 3.7a	264.5 ± 3.1a	284.1 ± 2.9a	229.6 ± 3.3a
2009	Control	232.0 ± 7.1a	269.7 ± 5.7a	227.1 ± 2.8b	180.6 ± 2.3b	199.3 ± 3.1a	228.1 ± 3.7a
	Low manure	232.2 ± 7.4a	271.6 ± 4.9a	231.2 ± 3.1b	181.0 ± 3.1b	199.7 ± 3.5a	229.4 ± 3.5a
	Medium manure	241.1 ± 6.6a	272.2 ± 3.7a	236.1 ± 4.6ab	189.7 ± 2.8a	202.3 ± 2.7a	229.4 ± 2.9a
	High manure	244.6 ± 6.9a	278.1 ± 5.2a	244.0 ± 4.9a	191.7 ± 3.3a	203.6 ± 3.3a	234.1 ± 3.2a
2010	Control	236.3 ± 3.1a	242.5 ± 4.3a	212.7 ± 2.9c	212.0 ± 3.7b	220.4 ± 2.8c	265.3 ± 6.7a
	Low manure	236.8 ± 3.7a	244.5 ± 3.9a	214.0 ± 3.1c	218.0 ± 4.1b	224.1 ± 3.3c	267.5 ± 5.7a
	Medium manure	238.6 ± 4.1a	245.6 ± 3.7a	224.4 ± 2.7b	245.9 ± 2.6a	236.7 ± 4.1b	271.7 ± 5.9a
	High manure	239.1 ± 2.7a	249.9 ± 4.7a	244.3 ± 3.5a	247.5 ± 3.3a	248.0 ± 3.7a	277.5 ± 6.3a

Values in the same column and same year followed by different letters indicate significant differences (Duncan p < 0.05).

**Table 2:** Effect of manure management on dry matter accumulation at different growth stages of maize.

Years	Treatments	Dry matter accumulation (g plant^−1^ )				
		Jointingstage	Big trumpetstage	Tasselingstage	Grain fillingstage	Maturitystage
2008	Control	1.3 ± 0.3a	16.9 ± 1.1d	92.7 ± 3.3d	168.8 ± 3.7d	216.8 ± 4.9d
	Low manure	1.3 ± 0.2a	19.0 ± 0.8c	109.1 ± 1.1c	178.6 ± 2.3c	248.5 ± 3.7c
	Medium manure	1.4 ± 0.3a	21.0 ± 0.5b	115.1 ± 3.2b	197.7 ± 3.1b	270.8 ± 4.6b
	High manure	1.4 ± 0.2a	23.4 ± 0.6a	124.3 ± 2.1a	210.0 ± 2.5a	297.9 ± 5.2a
2009	Control	1.4 ± 0.2a	18.0 ± 1.1d	87.1 ± 3.2d	165.1 ± 3.6c	218.4 ± 5.0c
	Low manure	1.5 ± 0.3a	20.7 ± 0.6c	105.8 ± 1.1c	178.9 ± 4.7b	238.0 ± 4.2b
	Medium manure	1.5 ± 0.2a	22.1 ± 0.3b	110.7 ± 1.7b	198.8 ± 4.4a	262.1 ± 7.2a
	High manure	1.5 ± 0.2a	24.9 ± 0.7a	120.8 ± 1.2a	209.3 ± 3.5a	276.3 ± 7.7a
2010	Control	1.5 ± 0.2a	14.9 ± 1.0d	90.3 ± 2.1d	166.1 ± 2.1c	200.5 ± 5.0c
	Low manure	1.5 ± 0.3a	21.7 ± 1.3c	100.8 ± 2.3c	173.7 ± 2.7b	223.8 ± 4.1b
	Medium manure	1.5 ± 0.2a	25.3 ± 0.9b	109.0 ± 3.1b	198.4 ± 3.7a	259.6 ± 6.3a
	High manure	1.6 ± 0.3a	28.5 ± 1.0a	117.6 ± 1.9a	203.6 ± 3.5a	271.3 ± 6.7a

Values in the same column and same year followed by different letters indicate significant differences (Duncan p < 0.05).

**Table 3:** Effect of manure management on rainfall use efficiency at different growth stages of maize.

Years	Treatments	Rainfall use efficiency (kg ha^−1^mm^−1^)				
		Seedling-jointingstage	Jointing-big trumpetstage	Big trumpet-tasselingstage	Tasseling-grainfilling stage	Grain filling-maturitystage
2008	Control	1.1 ± 0.2a	8.0 ± 0.2d	139.1 ± 3.1c	30.5 ± 0.5b	51.9 ± 2.1d
	Low manure	1.1 ± 0.1a	9.0 ± 0.2c	165.2 ± 4.1b	27.9 ± 1.2c	75.5 ± 1.5c
	Medium manure	1.2 ± 0.2a	10.6 ± 0.3b	172.5 ± 3.6b	33.1 ± 0.8a	79.0 ± 1.2b
	High manure	1.2 ± 0.1a	11.3 ± 0.2a	185.0 ± 3.1a	34.4 ± 1.0a	94.9 ± 1.3a
2009	Control	1.0 ± 0.2a	7.3 ± 0.2d	73.5 ± 2.7c	124.9 ± 1.5b	22.3 ± 0.6d
	Low manure	1.0 ± 0.1a	8.5 ± 0.3c	90.4 ± 2.2b	117.1 ± 1.7c	24.8 ± 0.5c
	Medium manure	1.0 ± 0.2a	9.1 ± 0.2b	94.1 ± 3.1b	141.2 ± 1.1a	26.5 ± 0.8b
	High manure	1.1 ± 0.1a	10.4 ± 0.3a	101.8 ± 0.9a	141.8 ± 1.2a	28.1 ± 1.0a
2010	Control	1.0 ± 0.1a	17.9 ± 2.1d	40.4 ± 0.2d	32.9 ± 2.3b	16.6 ± 2.5d
	Low manure	1.0 ± 0.1a	26.9 ± 1.7c	42.4 ± 0.3c	31.7 ± 1.1b	24.1±2.1c
	Medium manure	1.0 ± 0.2a	31.6 ± 1.0b	44.8 ± 0.7b	38.8 ± 1.2a	29.4 ± 1.2b
	High manure	1.0 ± 0.2a	35.9 ± 1.1a	47.7 ± 0.4a	37.3 ± 2.1a	32.6 ± 1.2a

Values in the same column and same year followed by different letters indicate significant differences (Duncan p<0.05).

**Table 4:** Rainfall in whole growth period of maize in the years of 2008–2010.

Years	Sowing-jointing stage	Rainfall (mm)				
		Jointing-bigtrumpet stage	Big trumpet-tasselingstage	Tasseling-grainfilling stage	Grain filling-maturitystage	Whole growthstage
2008	57.7	96.9	27.0	123.4	45.8	350.8
2009	71.5	111.8	46.6	30.9	118.3	379.1
2010	75.9	37.2	92.4	114.0	102.8	422.3
